# Green Biosynthesis and Characterization of Magnetic Iron Oxide (Fe_3_O_4_) Nanoparticles Using Seaweed (*Sargassum muticum*) Aqueous Extract

**DOI:** 10.3390/molecules18055954

**Published:** 2013-05-21

**Authors:** Mahnaz Mahdavi, Farideh Namvar, Mansor Bin Ahmad, Rosfarizan Mohamad

**Affiliations:** 1 Department of Chemistry, Faculty of Science, Universiti Putra Malaysia, 43400 UPM Serdang, Selangor, Malaysia; E-Mail: mahnaz.chem@gmail.com; 2 Department of Polymer Engineering, Faculty of Engineering, Shiraz Branch, Islamic Azad University, Shiraz 71993-3, Iran; 3 Institute of Tropical Forestry and Forest Products (INTROP), Universiti Putra Malaysia, 43400 UPM Serdang, Selangor, Malaysia; E-Mails: farideh.namvar@gmail.com (F.N.); farizan@biotech.upm.edu.my (R.M.); 4 Department of Medicine & Applied Biology Research Centre, Mashhad Branch, Islamic Azad University, Mashhad 917568, Iran; 5 Department of Bioprocess Technology, Faculty of Biotechnology and Biomolecular Sciences, Universiti Putra Malaysia, 43400 UPM Serdang, Selangor, Malaysia

**Keywords:** green synthesis, iron oxide nanoparticles, magnetic, *Sargassum muticum*

## Abstract

The synthesis of nanoparticles has become a matter of great interest in recent times due to their various advantageous properties and applications in a variety of fields. The exploitation of different plant materials for the biosynthesis of nanoparticles is considered a green technology because it does not involve any harmful chemicals. In this study, iron oxide nanoparticles (Fe_3_O_4_-NPs) were synthesized using a rapid, single step and completely green biosynthetic method by reduction of ferric chloride solution with brown seaweed (BS*, Sargassum muticum*) water extract containing sulphated polysaccharides as a main factor which acts as reducing agent and efficient stabilizer. The structural and properties of the Fe_3_O_4_-NPs were investigated by X-ray diffraction, Fourier transform infrared spectroscopy, field emission scanning electron microscopy (FESEM), energy dispersive X-ray fluorescence spectrometry (EDXRF), vibrating sample magnetometry (VSM) and transmission electron microscopy. The average particle diameter as determined by TEM was found to be 18 ± 4 nm. X-ray diffraction showed that the nanoparticles are crystalline in nature, with a cubic shape. The nanoparticles synthesized through this biosynthesis method can potentially useful in various applications.

## 1. Introduction

In recent years, novel size-dependent physicochemical properties have led to metallic iron nanoparticles of great potential in a wide range of applications, including magnetic storage media [1], ferrofluids [2], biosensors [3], catalysts [4], separation processes, and environmental remediation [5]. Specifically, magnetite (Fe_3_O_4_) is a common magnetic iron oxide having a cubic inverse spinel structure. The compound exhibits unique electric and magnetic properties based upon the transfer of electrons between Fe^2+^ and Fe^3+^ in octahedral sites [6].

According to their unique physical, chemical, thermal, and mechanical properties, and also by having suitable surface characteristics, superparamagnetic nanoparticles offer a great potential in many biomedical applications, such as cellular therapy, tissue repair, drug delivery, magnetic resonance imaging (MRI), hyperthermia, and magnetofection [7]. As magnetic particles accumulate in tumour tissues, they can play an important role in detection through MRI or electron microscopic imaging to locate and measure binding or as the drug carrier for certain anti-cancer drugs [8].

Currently, a large number of physical, chemical, biological, and hybrid methods are available to synthesize different types of nanoparticles [9]. The nanoparticles formed using each method show specific properties. However, biosynthesis of metal nanoparticles by plants is currently under development. Green nanotechnology has attracted a lot of attention and includes a wide range of processes that reduce or eliminate toxic substances to restore the environment. The synthesis of metal nanoparticles using inactivated plant tissue [10], plant extracts [11], exudates [12], and other parts of living plants [13] is a modern alternative for their production. Green synthesis of nanoparticles makes use of environmental friendly, non-toxic and safe reagents [14]. 

Historically, seaweed is a readily available food source that has been consumed by coastal communities likely since the dawn of time. Seaweed is consumed habitually in many countries in South-East Asia [15]. Marine algae refer to a wide variety of different species with different medicinal behavior, which are divided into two groups, namely microalgae and macroalgae. Marine macroalgae or seaweed, are plant-like organisms classified according to their pigmentation into green (chlorophytes), red (rhodophytes) and brown (phaeophytes). Seaweeds are well-known as functional food for their richness in lipids, minerals and certain vitamins, and also several bioactive substances like polysaccharides, proteins and polyphones, with potential medicinal uses against cancer [16], oxidative stress [17], inflammation [18], allergy [19], diabetes [20], thrombosis [21], obesity [22], lipidemia [23], hypertensive [24] and other degenerative diseases. Thus, their phytochemicals include hydroxyl, carboxyl, and amino functional groups, which can serve both as effective metal-reducing agents and as capping agents to provide a robust coating on the metal nanoparticles in a single step.

The current work describes a green and rapid method using brown seaweed (BS, *Sargassum muticum*) plant extract solution for the biosynthesis of iron oxide nanoparticles in ambient conditions, without any additive protecting nanoparticles from aggregating, template shaping nanoparticles or accelerants. The current simple synthetic green method using rapid precursors of BS plant extract provides high-yield nanosized materials with good optical properties, and the method can be used to prepare nanocrystalline oxides of other interesting materials. In this work, the characterization and formation mechanisms of Fe_3_O_4_-NPs are discussed. The Fe_3_O_4_-NPs were prepared using ferric chloride as iron precursor and BS extract as reducing agent and stabilizer.

## 2. Results and Discussion

### 2.1. Mechanism of the Fe3O4-NPs Formation in BS Extract

**Figure 1:**
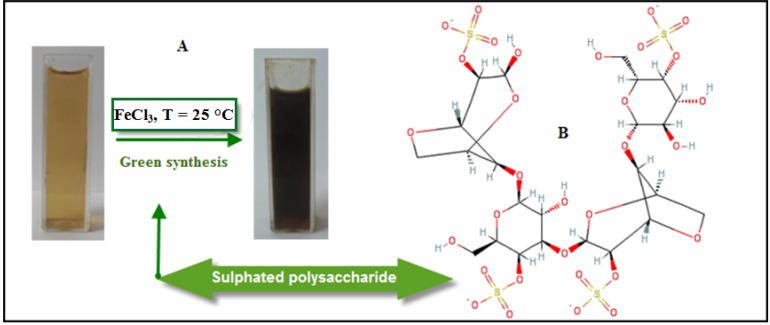
Photograph of synthesized Fe_3_O_4_-NPs in BS extract.

The improvement of reliable, nontoxic, and eco-friendly methods for synthesis of nanoparticles is of utmost importance to expand their biomedical applications [25]. The present work focused on the development of a biosynthetic method for the production of Fe_3_O_4_-NPs using brown seaweed (*Sargassum muticum*) extract. As shown in Figure 1A, the color of the Fe^3+^/BS extract solutions at room temperature rapidly changed from yellow to dark brown, indicating the formation of Fe_3_O_4_-NPs in the BS extract.

The addition of ferric chloride solution as iron precursor to the BS extract containing sulphated polysaccharides (Figure 1B) as a major component which has sulphate, hydroxyl and aldehyde group may cause the reduction of Fe^3+^ and stabilization of the nanoparticles. Decreasing in pH during the formation of Fe_3_O_4_-NPs signifies the involvement of the OH group in the reduction process. 

Initially, FeCl_3_ hydrolyzes to form ferric hydroxide and releases H^+^ ions thereafter; ferric hydroxide is partially reduced by the BS extract to form Fe_3_O_4_-NPs, while aldehyde groups are oxidized to the corresponding acids. The proposed green synthesis method for Fe_3_O_4_-NPs was found to be constructive and extremely reproducible. 

### 2.2. Characterization of Fe3O4Nanoparticles

#### 2.2.1. Infrared Spectroscopy

**Figure 2:**
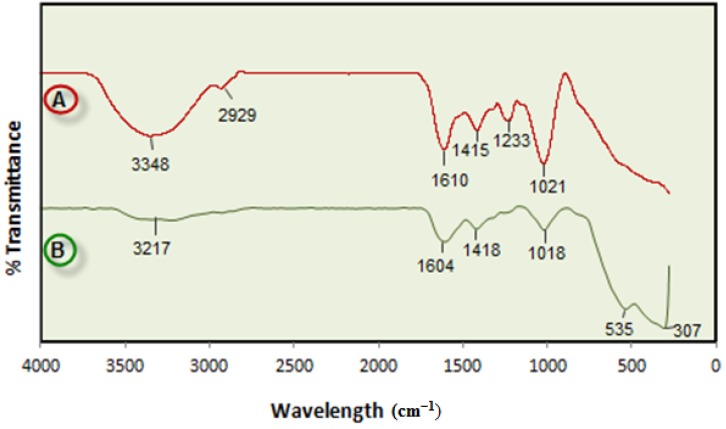
FT-IR spectrum for the BS powder (A) and Fe_3_O_4_-NPs from the biosynthesis reaction (B).

FTIR spectroscopy was used to identify the functional groups of the active components based on the peak value in the region of infrared radiation. Figure 2 shows FTIR spectra of BS powder and Fe_3_O_4_-NPs synthesized in BS extract. After complete bioreduction of iron ions, the BS extract was centrifuged for 2 min to isolate the Fe_3_O_4_-NPs from the compounds present in the solution.

The FTIR spectrum of BS powder has a characteristic stretching vibration band at 1,233 cm^−1^ denoting the asymmetric stretching vibration of the sulfate group, which disappeared after synthesis of Fe_3_O_4_-NPs indicated the involvement of sulfate group in the reduction process and the stabilization of Fe_3_O_4_-NPs. This is in agreement with [26], who reported that sulphated polysaccharides isolated from the marine alga *Porphyra vietnamensis* (Rhodophyta) had a strong ability to synthesize nanoparticles. It is also possible that the sulfate group play an important role in the reduction of metal ions by oxidation of aldehyde groups in the molecules to carboxylic acids [27].

The band at 1,021 cm^−1^ can be assigned to the symmetric C–O vibration associated with a C–O–SO_3_ group [28]. In addition, signals at 3,348 cm^−1^ (OH stretching) and 2,929 cm^−1^ (CH stretching) were also observed. After reduction of FeCl_3_ the decreases in intensity at 3,217 cm^−1^ imply the involvement of the OH group in the reduction process. The peak at 1,415 cm^−1^ indicates the C-C groups derived from aromatic rings that are present in the BS extract and also the peak at 1,610 cm^−1^ is attributed to the conjugated carbonyl (–C=O) group stretching vibration. The shift of the band from 1,610 cm^−1^ to 1,604 cm^−1^ was attributed to the binding of a C=O group with the nanoparticles [29]. The formation of Fe_3_O_4_ is characterized by two absorption bands at 535 and 307 cm^−1^ which correspond to the Fe–O bond in magnetite [30]. From the FTIR result, the soluble elements present in BS extract could have acted as capping agents preventing the aggregation of nanoparticles in solution, and thus playing a relevant role in their extracellular synthesis and shaping [31].

#### 2.2.2. X-ray Diffraction (XRD)

**Figure 3:**
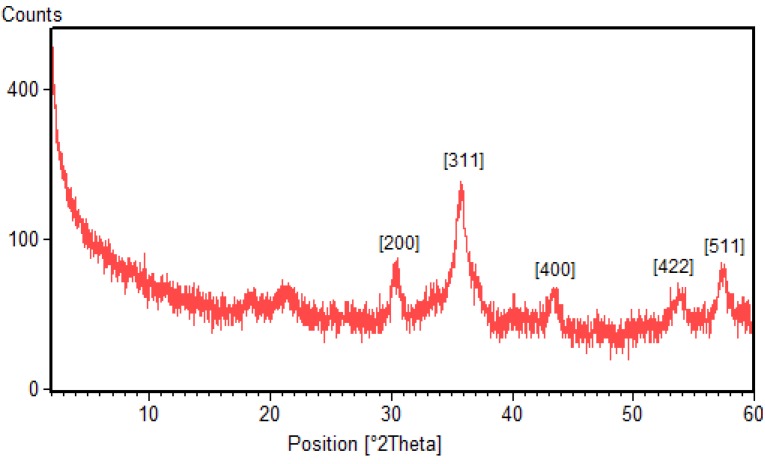
XRD pattern of Fe_3_O_4_-NPs synthesized using BS extract.

The phase identification and crystalline structures of the nanoparticles was characterized by X-ray powder diffraction. The X-ray diffraction patterns obtained for the Fe_3_O_4_-NPs synthesized using BS extract is shown in Figure 3. It is found that there exist strong diffraction peaks with 2θ values of 30.4°, 35.8°, 43.5°, 54.1° and 57.4°, corresponding to the crystal planes of (200), (311), (511) and (440) of crystalline Fe_3_O_4_-NPs, respectively. The results show the spinel phase structure of magnetite and are in agreement with the XRD standard for the magnetite nanoparticles [5].

The average particle sizes of Fe_3_O_4_-NPs can be estimated using the Debye-Scherrer equation, which gives a relationship between peak broadening in XRD and particle size that is demonstrated by following equation:

d = kλ/(β·cosθ)

where *d* is the particle size of the crystal, *k* is Sherrer constant (0.9), *λ* is the X-ray wavelength (0.15406 nm), β is the width of the XRD peak at half-height, and *θ* is the Bragg diffraction angle. Using the Scherrer equation the average crystallite sizes of the magnetic Fe_3_O_4_-NPs are found to be in the range of 17–25 nm.

#### 2.2.3. Morphology and Size Distribution of Nanoparticles

**Figure 4:**
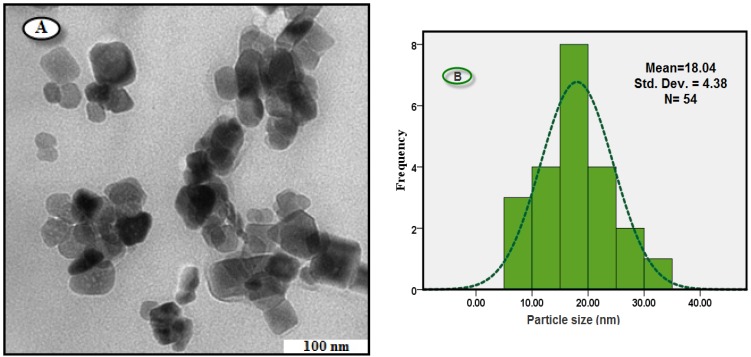
TEM image (**A**), and corresponding size distribution of Fe_3_O_4_-NPs synthesized using BS extract (**B**).

For TEM, a drop of the Fe_3_O_4_-NPs solution synthesized by treating ferric chloride solution with BS extract was deposited onto a TEM copper grid. After drying, the grid was imaged using TEM. The TEM image and the size distribution are shown in Figure 4A, Figure 4B. It is clear from Figure 4A that the sizes of Fe_3_O_4_-NPs are almost uniform, and all of the particles are cubic in shape. As shown in Figure 4B, the particle size distribution curve of Fe_3_O_4_-NPs indicated that mean diameter size of this nanoparticle was found to be 18 ± 4 nm. The XRD pattern suggested that the unassigned peaks may indicate the crystallization of bio-organic phase present in the extract which was also observed from TEM micrographs. The good correlation between particle sizes obtained from Scherrer equation and TEM supports the crystalline structure of the iron nanoparticles.

The morphology and structure of the Fe_3_O_4_-NPs were further investigated by field emission scanning electron microscopy (FESEM). 

**Figure 5:**
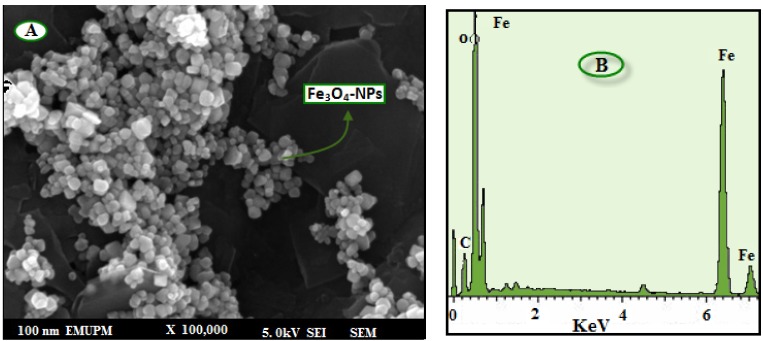
FESEM image (**A**) and energy-dispersive X-ray fluorescence spectrometry spectra of Fe_3_O_4_-NPs synthesized using BS extract (**B**).

The FESEM image and EDXRF spectra for the Fe_3_O_4_-NPs are shown in Figure 5. The FESEM image (Figure 5A) confirms that the Fe_3_O_4_-NPs are cubic in shape. In the EDXRF spectra (Figure 5B), the peaks around 0.8, 6.2, and 6.9 keV are related to the binding energies of Fe. Therefore, the EDXRF spectra for the Fe_3_O_4_/seaweed extract confirmed the presence of Fe_3_O_4_-NPs in the BS aqueous extract without any impurity peaks. 

#### 2.2.4. Ultraviolet-Visible Spectroscopy (UV-Vis)

**Figure 6:**
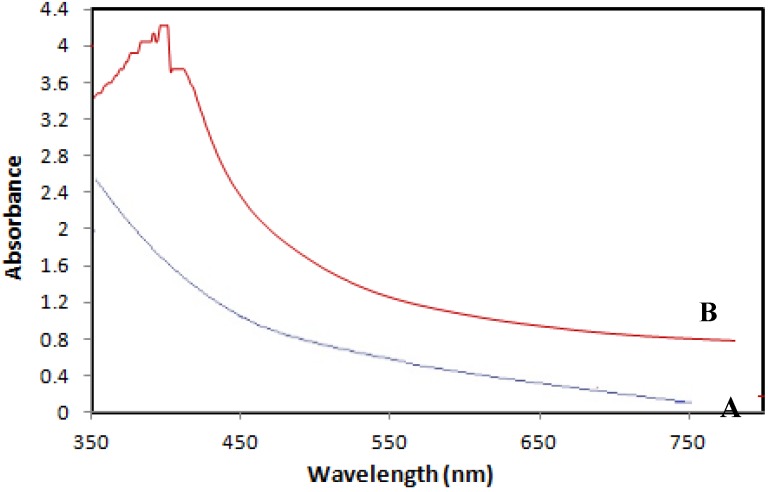
UV-visible absorption spectra of BS (A) and Fe_3_O_4_/BS extract (B).

Ultraviolet-visible spectroscopy (UV-Vis) refers to absorption spectroscopy in the UV-Visible spectral region. This means it uses light in the visible and adjacent (near-UV and near-infrared) ranges. The absorption in the visible range directly affects the perceived color of the chemicals involved. In this region of the electromagnetic spectrum, molecules undergo electronic transitions. The UV Visible spectrum of Fe_3_O_4_-NPs in the aqueous BS extract is shown in Figure 6. The two absorption peaks at wavelengths of 402 nm and 415 nm indicate the formation of iron nanoparticles.

#### 2.2.5. Vibrating Sample Magnetometry (VSM)

**Figure 7:**
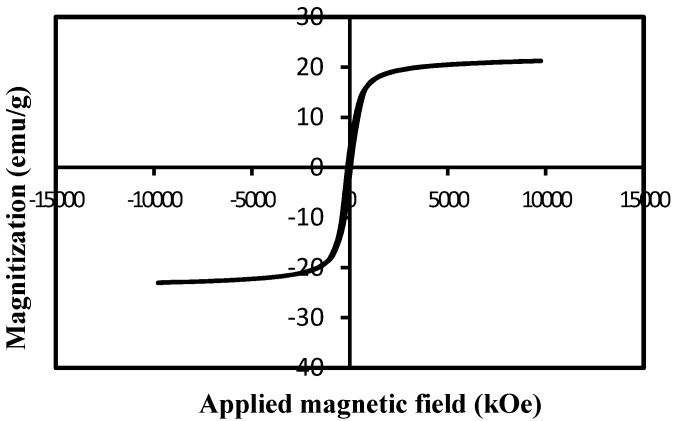
Magnitization curve of Fe_3_O_4_-NPs.

In order to study the magnetic behavior of Fe_3_O_4_-NPs, magnetization measurements recorded with VSM were performed. As can be observed in Figure 7, the specific saturation magnetization value was measured to be 22.1 emu/g for Fe_3_O_4_-NPs. The negligible coercivity Hc of hysteresis loop (82.3 Oe) and consequently no remanence Mr (2.75 emu/g) indicated the superparamagnetic nature of the Fe_3_O_4_-NPs. This could be due to the formed magnetic nanoparticles within the BS should be smaller than 25 nm, and they might be considered to have a single magnetic domain.

**Figure 8:**
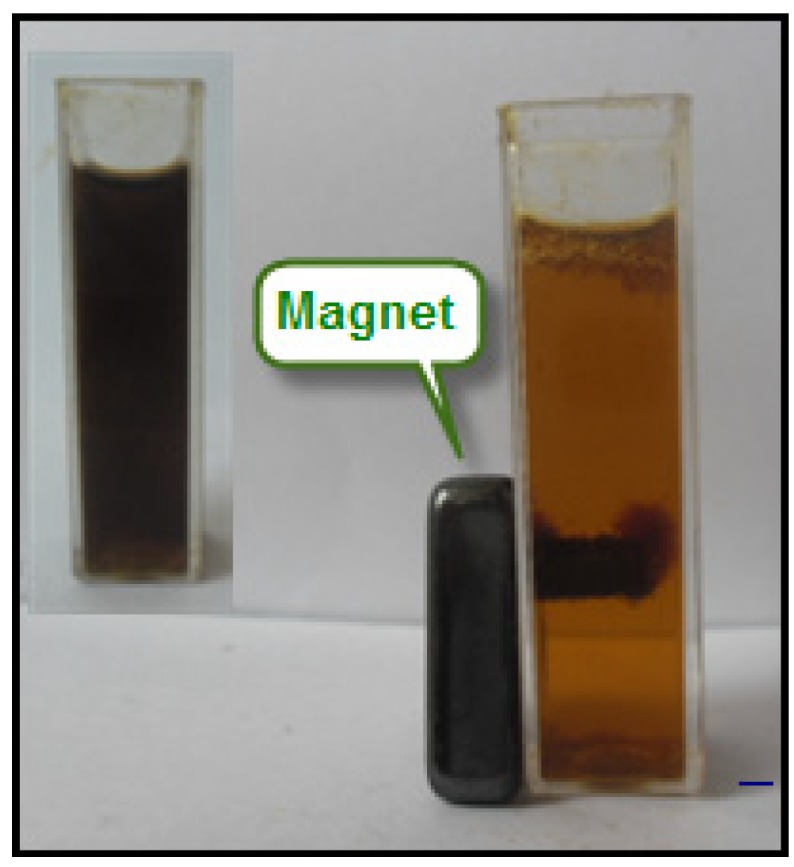
The separation of Fe_3_O_4_-NPs from BS extract under an external magnetic field.

The Fe_3_O_4_-NPs exhibited a magnetic property in the presence of a magnetic field. The Fe_3_O_4_-NPs were tested in BS extract by placing a magnet near the glass bottle as shown in Figure 8. The dark brown nanoparticles being attracted by a magnet and when the applied magnetic force is removed, the magnetic nanoparticles can easily be dispersed by simple shaking. Thus, the magnetic nanoparticles can be removed or recycled in the medium with a simple magnetic device.

All metal-reducing and stabilizer agents act as capping agent to provide a robust coating on the metal nanoparticles in a single step. Thus the magnetic property of this nanoparticles obtained with green synthesis is lower than nanoparticles which were synthesised by a coprecipitation method.

## 3. Experimental

### 3.1. Materials

Ferric chloride hexahydrate (FeCl_3_·6H_2_O, 98%) was purchased from Merck (Darmstadt, Germany) and was used without further purification. Specimens of the brown seaweed (BS, *Sargassum muticum*) were obtained from the coastal areas of Persian Gulf waters. All aqueous solutions were made using distilled deionized water (DDW). 

### 3.2. Extraction Preparation

**Figure 9:**
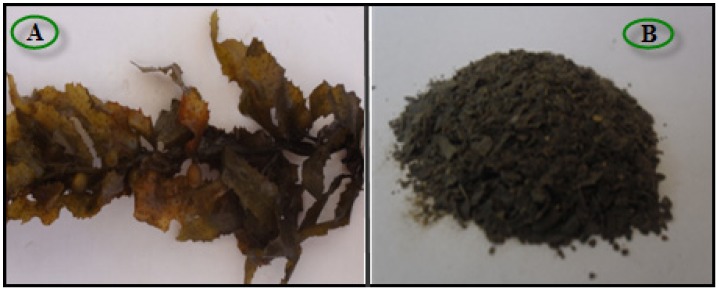
Brown seaweed (*Sargassum muticum*) (**A**), Brown seaweed (*Sargassum muticum*) powder (**B**).

The BS sample is shown in Figure 9. Specimens of BS were washed and stored at −20 °C (Figure 9A). For the production of extract, ground, freeze-dried seaweed samples (about 1 g, Figure 9B) were boiled with DDW (100 mL) in an Erlenmeyer flask while being continuously stirred for 15 min. The extract was cooled to room temperature, filtered, and stored at −20 °C before use.

### 3.3. Preparation of Fe3O4 Nanoparticles

Iron oxide nanoparticles (Fe_3_O_4_-NPs) were prepared by adding 0.1 M FeCl_3_ solution to the BS extract in a 1:1 volume ratio. Fe_3_O_4_-NPs were immediately obtained with the reduction process. The mixture was stirred for 60 min and then allowed to stand at room temperature for another 30 min. The obtained colloidal suspensions were then centrifuged and washed several times with ethanol and then dried at 40 °C under vacuum to obtain the Fe_3_O_4_-NPs.

### 3.4. Characterization Methods and Instruments

FT-IR spectra of the Fe_3_O_4_-NPs were recorded over the range of 400–4,000 cm^−1^ on a model spectrum 100 series (Perkin Elmer, Walthman, MA, USA) FTIR spectrophotometer. The crystalline structure and phase purity of the Fe_3_O_4_-NPs produced were identified by X-ray diffraction measurement (XRD-6000; Shimadzu, Tokyo, Japan). Transmission electron microscopy (TEM) observations were carried out on a Hitachi (Tokyo, Japan) H-7100 electron microscope with an acceleration voltage of 200 kV and the particle-size distributions were determined using the UTHSCSA Image Tool version 3.00 program. Field emission scanning electron microscopy (FESEM) was performed using a Philips (Eindhoven, The Netherlands) JSM-6360LA instrument to study the morphology of magnetic NPs. The energy dispersive X-ray fluorescence spectrometry (EDXRF) was carried out on a DX-700HS spectrometer (Shimadzu). The UV-visible spectra were recorded over the 300–700 nm range with a UV 1650 PC-Shimadzu B UV-visible spectrophotometer (Osaka, Japan). Magnetic properties of the samples were measured using a vibration sample magnetometer (VSM; Lake Shore Model 7400, (Tokyo, Japan) under magnetic fields up to 10 kOe.

## 4. Conclusions

A critical need in the field of nanotechnology is the development of reliable and ecofriendly processes for synthesis of metal oxide nanoparticles. Fe_3_O_4_-NPs with an average size of 18 ± 4 nm and cubic shapes were synthesized by bioreduction of ferric chloride solution with a green method using brown seaweed *(Sargassum muticum)* aqueous extract contain sulphated polysaccharides as the reducing agent and efficient stabilizer. The hydroxyl, sulphate and aldehyde group present in the BS extract are apparently involved in the bioreduction and stabilization of Fe_3_O_4_-NPs. The involvement of these groups in biosynthesis is revealed by FTIR analysis. The characteristics of the obtained Fe_3_O_4_-NPs were studied using FTIR, XRD, UV-visible, FESEM, EDXRF, TEM and VSM techniques. Biosynthesis of Fe_3_O_4_-NPs using green resources is a simple, environmentally friendly, pollutant-free and low-cost approach. Functional bioactivity of Fe_3_O_4_-NPs (antimicrobial) is comparably higher than particles which were synthesized by chemical method. This green method of synthesizing Fe_3_O_4_-NPs could also be extended to fabricate other, industrially important metal oxides.
